# An Analysis of the Last Clinical Encounter before Outpatient Mortality among Children with HIV Infection and Exposure in Lilongwe, Malawi

**DOI:** 10.1371/journal.pone.0169057

**Published:** 2017-01-18

**Authors:** Chris A. Rees, Robert J. Flick, David Sullivan, Menard Bvumbwe, Joseph Mhango, Mina C. Hosseinipour, Peter N. Kazembe

**Affiliations:** 1 Baylor College of Medicine Children’s Foundation Malawi, Lilongwe, Malawi; 2 Baylor College of Medicine, Houston, Texas, United States of America; 3 School of Medicine, University of Colorado, Denver, Colorado, United States of America; 4 University of North Carolina Project-Malawi, Lilongwe, Malawi; 5 Baylor International Pediatric AIDS Initiative at Texas Children's Hospital, Houston, Texas, United States of America; Central University of Tamil Nadu, INDIA

## Abstract

**Background:**

Human immunodeficiency virus (HIV) contributes to nearly 20% of all deaths in children under five years of age in Malawi. Expanded coverage of antiretroviral therapy has allowed children to access treatment on an outpatient basis. Little is known about characteristics of the final outpatient encounter prior to mortality in the outpatient setting.

**Methods:**

This retrospective cohort study assessed clinical factors associated with mortality among HIV-exposed infants and HIV-infected children less than 18 years of age at the Baylor College of Medicine Abbott Fund Children’s Center of Excellence in Lilongwe, Malawi. We compared clinical indicators documented from the final outpatient encounter for patients who died in the outpatient setting versus those who were alive after their penultimate clinical encounter.

**Results:**

Of the 8,546 patients who were attended to over a 10-year period at the Baylor Center of Excellence, 851 had died (10%). Of children who died, 392 (46%) were directly admitted to the hospital after their last clinical encounter and died as inpatients. Of the remaining 459 who died as outpatients after their last visit, 53.5% had a World Health Organization (WHO) stage IV condition at their last visit, and 25% had a WHO stage III condition. Multivariate regression analysis demonstrated that poor nutritional status, female gender, shorter time as a patient, more clinical encounters in the prior month, if last visit was an unscheduled sick visit, and if the patient had lost weight since their prior visit independently predicted increased mortality in the outpatient setting after the final clinical encounter.

**Conclusion:**

Clinical indicators may assist in identifying children with HIV who have increased risk of mortality in the outpatient setting. Recognizing these indicators may aid in identifying HIV-infected children who require a higher level of care or closer follow-up.

## Introduction

Human immunodeficiency virus (HIV) is a leading cause of morbidity and mortality among children in sub-Saharan Africa. In Malawi, the expansion of antiretroviral therapy (ART) has been associated with a decline in under-five mortality from 244 to 71 per 1,000 [1,2]. However, HIV still contributes to nearly 20% of all deaths in children under five in Malawi [3].

Several clinical factors contribute to mortality in children with HIV. Young age contributes to mortality as those who are not identified and started on treatment in the first two years of life have an increased risk of mortality [4]. There is also a high estimated prevalence of tuberculosis among children with HIV [5]. Potentially life-threatening malignancies, such as Kaposi sarcoma and non-Hodgkin lymphoma, are also more common in children living with HIV than in the general pediatric population in sub-Saharan Africa [6]. Malnutrition and pneumonia contribute to a large proportion of inpatient mortality among children in Malawi [7]. Moreover, low socioeconomic status increases the risk of mortality among patients living with HIV [8].

Targeted interventions have shown decreased inpatient mortality among children in Malawi through improved triage systems [9,10] and routine inpatient provider-initiated HIV testing and counseling leading to earlier detection of HIV [11]. Despite such efforts, inpatient mortality rates remain as high as 5–7% [12].

While factors associated with inpatient mortality have been described, little is known about children with, or exposed to, HIV who die in the outpatient setting after clinical encounters. A more complete understanding of factors associated with outpatient mortality could reduce mortality by identifying children requiring closer follow-up or acute care. Our aim was to describe characteristics of the last outpatient visit among HIV-infected and HIV-exposed children who died in the outpatient setting in Lilongwe, Malawi.

## Methods

### Study Setting

This study was conducted at the Baylor College of Medicine Abbott Fund Children’s Center of Excellence (Baylor-Malawi), the largest pediatric HIV clinic in Malawi. Founded in 2005, Baylor-Malawi provides comprehensive care for HIV-infected and HIV-exposed infants and children in the Lilongwe area [13]. It is a national referral center for complex pediatric HIV cases. As of June 2015, 8,546 patients had been seen for 183,831 individual encounters at Baylor-Malawi. Similar to other health care settings in Malawi, clinical officers and nurse clinicians provide the majority of care at Baylor-Malawi [14].

### Study Design

This was a retrospective study of all HIV-infected and HIV-exposed children less than 18 years of age. PubMed was used to conduct the literature review that framed this study. The following key words were used to find relevant articles: “pediatric” or “childhood”, “mortality”, “inpatient”, “outpatient”, “HIV”, “Malawi”, and “Africa”. Studies were included if they included relevant data. Data were abstracted from the Baylor-Malawi electronic medical record for analysis from clinical encounters that occurred between January 1, 2005 and June 5, 2015. The last visit before death was used for patients with known death. The patient’s death was discovered by clinic staff when patients did not return for scheduled follow up appointments and phone calls or home visits were made to find patients. Specific location (i.e. home, health center, etc.) of the patient’s death was not routinely provided by caregivers. For patients recorded as alive, one visit prior to their last recorded visit was used for analysis.

Exclusion criteria included age over eighteen years at last visit, not exposed to HIV, or HIV-uninfected confirmed by two consecutive antibody tests. Furthermore, patients whose last visit resulted in immediate hospitalization and death before discharge were also excluded. This was done to exclude patients for whom clinicians appropriately triaged to a higher level of care, thereby better assessing risk factors for death among patients who were sent home.

Nutritional status was generated based on Malawi’s Guidelines for the Clinical Management of HIV in Children and Adults, which defines severe malnutrition having weight for height scores of 70% or less or a middle upper arm circumference of <12 centimeters for children 0–14 years of age. Moreover, for children 15 years of age and older, a body mass index <16.0 was used to define severe malnutrition [15]. The authors CAR and DS reviewed all charts of patients who were documented as deceased without a hospital admission to ascertain descriptive data including clinical World Health Organization (WHO) staging at the last clinical encounter, documented diagnosis during the final clinical encounter, and whether the clinician documented a guarded prognosis. These data were extracted from the electronic health record and recorded in an Excel spreadsheet. Cluster of differentiation 4 (CD4) counts were performed as part of routine clinical care at Baylor-Malawi using the BD FACSCount^™^ machine and viral loads were processed at the Kamuzu Central Hospital laboratory using the Abbott M2000 sp machine^™^. Documented CD4 counts and viral loads from the six-month period prior to the final clinical encounter was used for analysis.

### Statistical Analysis

Non-parametric variables were compared using the Wilcoxon rank-sum test, and categorical variables using X^2^ or Fisher’s exact tests. Binary logistic regression was used to calculate unadjusted odds ratios with 95% confidence intervals (95% CI). Multivariate logistic regression models were constructed using backward elimination. The initial model incorporated all covariates with p < 0.10 in bivariate analysis. Covariates were then eliminated in a stepwise fashion based on a preset alpha level of 0.05. The Hosmer-Lemeshow goodness-of-fit test was used to assess the final model. All analyses were done in Stata Special Edition 14.1 (Statcorp; College Station, TX). The National Health Sciences Research Council of Malawi and the Baylor College of Medicine Institutional Review Board approved the extraction of data from the electronic medical record at Baylor-Malawi. As this study was retrospective, informed consent was not obtained for the review of medical records. Data were anonymized and de-identified prior to analysis to maintain confidentiality.

## Results

**Fig 1:**
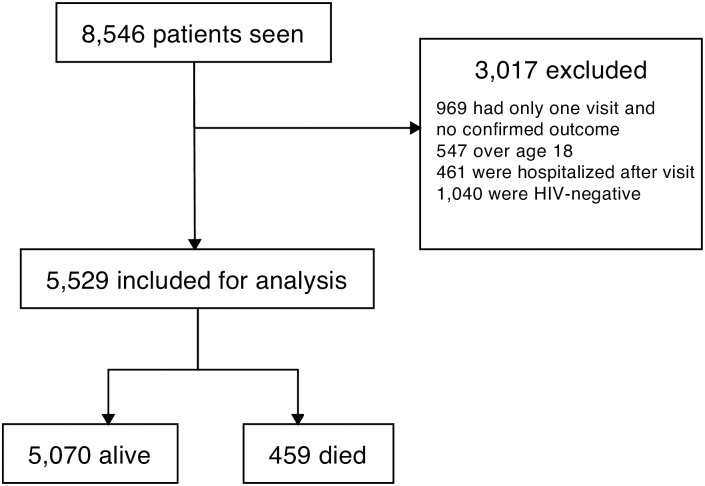
Consort diagram demonstrating exclusion criteria and patient population selection.

During the ten-year period analyzed in this study, there were a total of 8,546 patients seen at Baylor-Malawi (7,695 alive, 851 dead). Of these, 3,017 were excluded, with the remaining 5,529 included for analysis. Of these, 459 (8.3%) died as outpatients, and 5,070 (91.7%) were alive (Fig 1). The majority of patients who died in the outpatient setting had a WHO stage IV condition (53.5%), while a quarter of them had a WHO stage III condition (Table 1). The most common WHO stage IV conditions encountered in this population were severe acute malnutrition, Kaposi sarcoma, and esophageal thrush. Seventeen percent of patients who died in the outpatient setting after their last clinical encounter were being treated for pulmonary tuberculosis. Ten percent of the patients who died were exposed infants. The most commonly encountered visit diagnoses before outpatient mortality were pneumonia, gastroenteritis, and malaria, though 10.7% of patients who died after the last encounter were said to have been doing well and had no acute diagnosis. Documentation of a guarded prognosis was found in only 12.2% of the charts of patients who died in the outpatient setting.

**Table 1:** Descriptive clinical characteristics of patients who died in the outpatient setting after their last clinical encounter.

Characteristic	n (%)
**Exposed infant**	46 (10.0)
WHO stage*	
•I	43(14.9)
•II	18 (6.3)
•III	73 (25.3)
•IV	154 (53.5)
WHO stage condition*	
•Pulmonary tuberculosis	81 (17.6)
•Oral/esophageal thrush	67 (14.6)
•Severe acute malnutrition	62 (13.5)
•Kaposi sarcoma	47 (10.2)
•Moderate malnutrition	42 (9.2)
•Pneumocystis pneumonia	8 (1.7)
**Most common documented diagnoses at last visit**	
•Pneumonia	50 (10.9)
•Doing well	49 (10.7)
•Gastroenteritis	35 (7.6)
•Malaria	27 (5.9)
**Prognosis documented as guarded or poor**	
•No	403 (87.8)
•Yes	56 (12.2)

*Excludes 25 with missing documented WHO staging in electronic medical record and 46 exposed infants.

Bivariate analysis revealed that patients who died in the outpatient setting after the last clinical encounter were younger, had been patients at Baylor-Malawi for less time, and had more visits in the last 30 days before their final encounter than patients who survived. The final encounter was more frequently an unscheduled sick visit for patients who died than for patients who were alive (Table 2). Patients who died were also more likely to have missed their penultimate clinical appointment.

**Table 2:** Characteristics of visits among patients who died as outpatients compared to patients still alive after their last clinical encounter at Baylor-Malawi.

	Bivariate Analysis						Multivariate Analysis	
Characteristic	Alive (N = 5070)		Died (N = 459)		Odds Ratio (95% CI)	p-value	Adjusted Odds Ratio (95% CI)	p-value
**Median time as patient in years (IQR)**	1.84	(0.40–5.2)	0.3	(0.07–1.1)	0.65 (0.61–0.70)	<0.0001	0.77 (0.72–0.82)	<0.001
**Median time since last visit in months (IQR)**	1.2	(0.9–2.0)	0.6	(0.3–1.2)	1.03 (1.01–1.04)	<0.0001		
**Number of visits in 30 day period prior to last visit**	0	(0–1)	1	(0–2)	2.06 (1.86–2.29)	<0.001	1.49 (1.31–1.69)	<0.001
**Unscheduled sick visit—n (%)**								
•No	4,770	(94.1)	384	(83.7)	*Referent*		*Referent*	
•Yes	300	(5.9)	75	(16.3)	3.11 (2.36–4.08)	<0.0001	1.46 (1.31–1.69)	<0.001
**Missed last visit—n (%)**								
•No	3538	(69.8)	353	(76.9)	*Referent*	<0.0001		
•Yes	286	(5.6)	42	(9.2)	1.47 (1.05–2.07)			

Patients who died in the outpatient setting were more likely to have lost weight since their penultimate clinical encounter and to have any form of malnutrition with severe acute malnutrition being most predictive of mortality (Table 3). Patients had a five-fold increased risk of dying in the outpatient setting if they were on medications for tuberculosis at their last clinical encounter. Clinical instability denoted by lab values, namely a detectable viral load or a CD4 count below 200 in the six-month period prior to the final clinical encounter also predicted death.

**Table 3:** Clinical characteristics among patients who died as outpatients compared to matched patients who were alive after their last clinical encounter at Baylor-Malawi.

Characteristic	Bivariate Analysis						Multivariate Analysis	
	Alive (N = 5070)		Died (N = 459)		Odds Ratio (95% CI)	p-value	Adjusted Odds Ratio (95% CI)	p-value
**Median age at last visit in years (IQR)**	6.8	(2.6–11.1)	2.3	(1.0–7.6)	0.89 (0.87–0.91)	<0.0001		
**Gender—n (%)**								
•Male	2560	(50.5)	254	(55.3)	*Referent*			
•Female	2510	(49.5)	205	(44.7)	0.82 (0.68–0.99)	0.047	0.75 (0.59–0.96)	0.02
**Nutritional status at last visit—n (%)**								
•Within normal limits	4492	(94.9)	223	(61.1)	*Referent*	<0.0001	*Referent*	
•Mild	73	(1.5)	16	(4.4)	4.41 (2.53–7.71)		2.55 (1.40–4.65)	0.002
•Moderate	113	(2.4)	56	(15.3)	9.98 (7.05–14.13)		4.71 (3.22–6.88)	<0.001
•Severe	53	(1.1)	70	(19.2)	26.6 (18.17–38.96)		10.83 (7.17–16.37)	<0.001
**Weight loss since last visit—n (%)**								
•No	3996	(78.8)	276	(60.1)	*Referent*	<0.0001	*Referent*	
•Yes	1074	(21.2)	183	(39.9)	2.47 (2.02–3.01)		2.33 (1.82–3.00)	<0.001
**On antiretroviral therapy**								
•No	1537	(30.3)	168	(36.6)	*Referent*	<0.0001		
•Yes	3353	(66.1)	241	(52.5)	0.66 (0.54–0.81)			
**Antiretroviral therapy regimen**								
•Nevirapine-based (1st line)	2907	(86.9)	217	(90.4)	*Referent*	0.112		
•Protease inhibitor-based (2nd line)	440	(13.1)	23	(9.6)	0.7 (0.45–1.09)			
**On tuberculosis treatment at last visit**								
•No	4937	(97.4)	405	(88.2)	*Referent*		*Referent*	
•Yes	131	(2.6)	54	(11.8)	5.02 (3.60–7.01)	<0.0001	1.98 (1.29–3.04)	0.002
**CD4 Count**								
•<50	86	(1.7)	40	(8.7)	6.15 (4.15–9.11)	<0.0001		
•50–99	56	(1.1)	24	(5.2)	5.67 (3.46–9.27)			
•100–199	140	(2.8)	31	(6.8)	2.93 (1.95–4.40)			
•200–349	402	(7.9)	33	(7.2)	1.09 (0.75–1.58)			
•350–499	499	(9.8)	37	(8.1)	0.98 (0.69–1.40)			
Over 500	3887	(76.7)	294	(64.1)	*Referent*			
**Detectable viral load**								
•No	1541	(71.1)	30	(38.5)	*Referent*	<0.0001		
•Yes	626	(28.9)	48	(61.5)	3.94 (2.47–6.27)			

Unadjusted and adjusted odds ratios were calculated for clinical characteristics predicting outpatient mortality among patients with HIV (Table 3). ART status was omitted because of collinearity. Time on ART, missed last visit, pill count, and adherence indicators were also omitted in multivariate regression due to the frequency of missing values. The following variables predicted death in the final model: poor nutritional status, female gender, shorter time as a patient, more clinical encounters in the prior month, if last visit was an unscheduled sick visit, and if the patient had lost weight since their prior visit.

## Discussion

This study aimed to identify significant clinical factors present during the last clinical encounter prior to mortality in the outpatient setting to inform clinicians about key warning signs and symptoms. Clinicians should recognize these indicators and carefully consider the appropriate disposition of the patient in settings similar to Malawi where health care is often limited.

Severe acute malnutrition and weight loss prior to the final clinical encounter predicted mortality in the outpatient setting in this study. Malnutrition is a well-documented cause of death among children living with HIV in Malawi [16]. Though the WHO has clear indications for inpatient admission for nutritional rehabilitation [17], Malawi’s guidelines for inpatient admission for malnutrition are based on modified criteria from the National Center for Health Statistics [18]. Nevertheless, studies have shown that HIV-infected children have a threefold higher mortality rate while undergoing inpatient nutritional rehabilitation when compared to children without HIV [19,20]. Our results extend that finding and suggest that any form of malnutrition among HIV-infected and HIV-exposed children increased their risk of outpatient mortality. Thus, HIV-infected children with comorbid malnutrition who do not meet admission criteria should be closely followed in the outpatient setting at a minimum with supplementary ready-to-use therapeutic food, and potentially may need routine inpatient rehabilitation. Unfortunately, one study in Malawi showed that as many as 18.5% of children die at home after hospital admission for severe acute malnutrition [16]. Home-based therapy with community health workers should also be considered as one study from Malawi showed that ready-to-use therapeutic food administered by village health aides is an effective approach to treating malnutrition during food crises in areas lacking health services [21].

Other studies have shown that many children with HIV in Africa present late in the course of their illness and are oftentimes diagnosed when admitted to the hospital [22, 23]. Similarly, in this study, patients who died in the outpatient setting after their last clinical encounter were more frequently new or unestablished patients. This may be due, in part, to Baylor-Malawi serving as a referral center for children with complicated cases of HIV or due to late referral. Moreover, late diagnosis, which has been shown to be associated with increased mortality among children with HIV in similar settings [24], may have also contributed to this observed phenomenon. Furthermore, patients who died in the outpatient setting were more likely to have more clinical encounters in the month preceding their final encounter, which may serve as a warning sign to clinicians when attending to these patients.

Tuberculosis is estimated to be the leading cause of death among children with HIV worldwide [25]. Previous studies have indicated mortality rates to be as high as 20.2% among children with HIV who develop tuberculosis [26]. A meta-analysis assessing post-mortem diagnosis of tuberculosis among children and adults with HIV showed that 45.8% of cases of tuberculosis remained undiagnosed at the time of death [27]. Similar to the results of a retrospective cohort study conducted in Southern Malawi [28], the present study’s results indicate that the presence of clinically diagnosed tuberculosis at the final clinical encounter predicted mortality in the outpatient setting.

Three out of four children who died in this study had a WHO stage III or IV condition during their last clinical encounter. The association of advanced HIV disease and mortality has been documented in other studies as well [28]. The results from this study raise the question of whether serious conditions in HIV-infected and exposed children are under recognized or, alternatively, if children with HIV who are relatively clinically stable can rapidly deteriorate. One study from Malawi showed that clinicians in outpatient settings failed to diagnose nearly 40% of cases of severe pneumonia according to the WHO’s Integrated Management of Childhood Illnesses guidelines [14]. While such guidelines are designed to be clinically driven, other limitations, such as limited laboratory testing, create challenges for accurate diagnoses in resource-limited settings. For example, blood cultures are not routinely available in Malawi and the utility of sputum testing for tuberculosis in children is sub-optimal [29]. These limitations force clinicians to rely on clinical acumen and minimal laboratory data to make critical diagnoses, with consistently high rates of discordance between clinical and postmortem diagnoses by autopsy [30].

### Limitations

This retrospective study has several limitations. Some data were incomplete as all variables were abstracted from routinely collected information contained in the electronic health records and thus our results may not entirely reflect the clinical indicators present during the final clinical encounter prior to outpatient mortality. Moreover, malnutrition was documented based on the clinician’s assessment using Malawi’s guidelines which changed over the study period. Specifically, there was a transition towards the use of body mass index and z-score charts in 2012, which may detect more children with malnutrition requiring inpatient rehabilitation. Moreover, middle upper arm circumference was not recorded for all patients, which is potentially more sensitive in detecting the need for admission among malnourished children [31]. Patients were identified as having died in the outpatient setting when their following appointment had been missed and clinic staff made phone calls or home visits to find the patient, thus, the cause of death was rarely available. Verbal autopsies may elucidate the cause of death in these patients and merits further investigation. Moreover, the exact date of death was not available and thus the authors were unable to assess how the aforementioned clinical indicators relate to time and mortality in the outpatient setting. Lastly, while our aim was to identify patients who died in the outpatient setting without being admitted, it is possible that some of the patients who died presented to a hospital without the advice of a clinician at Baylor-Malawi. Nonetheless, our results show stark differences in the final clinical encounters of patients who died and those who did not.

## Conclusions

Poor nutritional status, female gender, shorter time as a patient, more clinical encounters in the prior month, if last visit was an unscheduled sick visit, and if the patient had lost weight since their prior visit are clinical indicators which predicted increased outpatient mortality after the final clinical encounter among children with or exposed to HIV in Lilongwe, Malawi. Identifying these indicators may aid in the recognition of the need for admission or, at a minimum, close outpatient follow-up. Future studies are needed to develop and validate risk stratification scores to further aid in the identification of children with, or exposed to, HIV at risk for outpatient mortality. Moreover, future programs assigning community health workers to patients with these indicators may reduce outpatient mortality among children with HIV in settings similar to Malawi.
